# DNA vaccine containing the mycobacterial hsp65 gene prevented insulitis in MLD-STZ diabetes

**DOI:** 10.1186/1476-8518-7-4

**Published:** 2009-09-15

**Authors:** Rubens R Santos, Alexandrina Sartori, Deison S Lima, Patrícia RM Souza, Arlete AM Coelho-Castelo, Vânia LD Bonato, Célio L Silva

**Affiliations:** 1 University of São Paulo, Ribeirão Preto Medical School, Department of Biochemistry and Immunology, Ribeirão Preto, São Paulo, Brazil; 2 Department of Clinical Analyses, School of Pharmaceutical Sciences, São Paulo State University, Araraquara, São Paulo, Brazil; 3 Bioscience Institute, São Paulo State University, Botucatu, São Paulo, Brazil

## Abstract

**Background:**

Our group previously demonstrated that a DNA plasmid encoding the mycobacterial 65-kDa heat shock protein (DNA-HSP65) displayed prophylactic and therapeutic effect in a mice model for tuberculosis. This protection was attributed to induction of a strong cellular immunity against HSP65. As specific immunity to HSP60 family has been detected in arthritis, multiple sclerosis and diabetes, the vaccination procedure with DNA-HSP65 could induce a cross-reactive immune response that could trigger or worsen these autoimmune diseases.

**Methods:**

In this investigation was evaluated the effect of a previous vaccination with DNA-HSP65 on diabetes development induced by Streptozotocin (STZ). C57BL/6 mice received three vaccine doses or the corresponding empty vector and were then injected with multiple low doses of STZ.

**Results:**

DNA-HSP65 vaccination protected mice from STZ induced insulitis and this was associated with higher production of IL-10 in spleen and also in the islets. This protective effect was also concomitant with the appearance of a regulatory cell population in the spleen and a decreased infiltration of the islets by T CD8^+ ^lymphocytes. The vector (DNAv) also determined immunomodulation but its protective effect against insulitis was very discrete.

**Conclusion:**

The data presented in this study encourages a further investigation in the regulatory potential of the DNA-HSP65 construct. Our findings have important implications for the development of new immune therapy strategies to combat autoimmune diseases.

## Background

Type 1 diabetes is an autoimmune disorder characterized by destruction of the insulin-producing β cells found in the pancreatic islets of Langerhans [1,2]. This condition manifests as a chronic inflammatory response involving islet infiltration (insulitis) by lymphocytes and monocytes. In susceptible strains of mice, an inflammatory form of diabetes with clinical and immunohistological features similar to those of human type 1 diabetes mellitus can be induced by injections with multiple low doses of streptozotocin (STZ) [3,4]. The majority of these mice develop hyperglycemia within 2-3 weeks after STZ injections [3,4].

The primary mediators of β-cell destruction are CD4^+ ^and CD8^+ ^T cells [5,6]. Pathogenic CD4^+ ^T cells typically exhibit a T-helper (Th)-1 cell phenotype, characterized by increased secretion of interferon gamma (IFN-γ) and tumor necrosis factor alpha (TNF-α). In addition, a number of β cell autoantigens, including insulin, islet antigen 2, and glutamic acid decarboxylase 65 (GAD65) have been identified as targets of CD4^+ ^Th1 cells [7]. Furthermore, Cohen et al., showed that, early in the process of β-cell destruction, nonobese diabetic (NOD) mice spontaneously developed antibodies and T cells reactive to murine 60-kDa heat shock protein (Hsp60) [8,9], which is cross-reactive with the mycobacterial Hsp65 molecule [10].

Interestingly, administration of β cell auto-antigens or derived peptides can prevent initiation of the disease process or even suppress established β cell autoimmunity under certain conditions in mice [11-16]. In many of these studies, protection is mediated through the induction of β cell-specific regulatory T cells. Th2 and/or Th3 cells suppress Th1 cell differentiation through a bystander mechanism involving secretion of regulatory cytokines including interleukin (IL)-4, IL-10, and transforming growth factor beta (TGF-β) [17,18]. These cytokines either act directly on naive T cells or modulate the function of antigen-presenting cells.

A great deal of interest has been focused on the use of plasmid DNA to elicit cellular and humoral immunity in the context of infectious diseases and cancer immunotherapy [19,20]. Combinations of plasmid DNAs encoding antigens and different cytokines can be used to influence the nature and magnitude of the immune response. They are used, for example, to prevent autoimmunity in various models [21-23]. Cohen et al., showed that vaccination with a DNA construct encoding human Hsp60 inhibited diabetes in NOD mice. Prevention of diabetes was associated with decreased insulitis, and down-regulation of the proliferative T-cell response to Hsp60 [24].

Our group has explored the prophylactic and therapeutic potential of a DNA vaccine containing the gene of a heat shock protein from *Mycobacterium leprae *(DNA-HSP65) in experimental tuberculosis [25-28]. We also observed that this vaccine was protective against experimental pristane-induced arthritis [29] and spontaneous diabetes in nonobese diabetic mice [30]. In the arthritis model this effect was associated with down-regulation in IL-6 and IL-12 production and up-regulation of the anti-inflammatory cytokine IL-10 [29]. Moreover, the protection in NOD mice was associated with a clear shift in the cellular infiltration pattern in the pancreas and an increased staining for IL-10 in the islets [30].

Within this context, the present study was designed to investigate the ability of the DNA-HSP65 vaccine to modulate the development of STZ-induced diabetes in C57BL/6 mice. In addition, we investigated the mechanisms associated with the immune modulation that DNA-HSP65 injection elicited in the spleen and pancreas. Our results show that either the vector (DNAv) alone or DNA carrying the gene encoding Hsp65 had no deleterious effect on the course of STZ-induced diabetes. In addition, the administration of DNA-HSP65 prior to STZ injection, partially avoided the development of destructive insulitis. DNA-HSP65-injected mice presented a pronounced increase in the number of CD4/CD25^+ ^cells in the spleen and decreased CD8^+ ^cellular infiltrates in the pancreas. In the spleen and pancreas of DNA-HSP65-injected mice, levels of IL-10 were higher and levels of TNF-α were lower than those observed in the control groups (not injected or DNA inoculated mice). Indeed, the protective effect of DNA-HSP65 injection in STZ-induced diabetes seemed to be related to both: a non specific component constituted by the vector and a specific component represented by the activation of regulatory T cells specific for HSP65, since the degree of protection evoked by the vaccine was comparatively higher.

## Materials and methods

### Animals

C57BL/6 male specific pathogen-free mice, 6-8 weeks old, were bred in the Animal Facility of the University of São Paulo at Ribeirão Preto Medical School. The animals were maintained on a 12-hour light/dark cycle and were given free access to food and autoclaved water. Each experiment was performed in triplicate. The following six groups of mice were studied: control-injected with phosphate-buffered saline (PBS); DNA-HSP65 - injected with the DNA-HSP65 vaccine; DNAv - injected with the empty vector; STZ - injected with STZ on 5 consecutive days; DNA-HSP65+STZ - injected with DNA-HSP65 prior to STZ; DNAv+STZ - injected with DNAv prior to STZ.

### Plasmid purification and immunization protocol

The DNA-HSP65 vaccine was derived from the pcDNA3 plasmid (Invitrogen, Carlsbad, CA), which had been previously digested with *Bam*HI and *Not*I (Gibco BRL, Gaithersburg, MD) by inserting a 3.3-kb fragment corresponding to the *M. leprae *Hsp65 gene and the cytomegalovirus intron A [31]. The empty pcDNA3 plasmid was used as a control. Luria-Bertani liquid medium (Gibco BRL) containing ampicillin (100 μg/ml) was used to culture DH5α *Escherichia coli*, transformed either with pcDNA3 plasmid or with the plasmid containing the Hsp65 gene (DNA-HSP65). Plasmids were purified using the Concert High Purity Maxiprep System (Gibco BRL). Plasmid concentration was determined by spectrophotometry at λ = 260 and at λ = 280 nm using the Gene Quant II apparatus (Pharmacia Biotech, Cambridge, UK). Mice were intramuscularly injected with three doses (100 μg each) of DNA-HSP65 or DNAv given at 2-week intervals. Control animals were injected with PBS.

### STZ-induced diabetes and analysis of glycemic levels

In order to induce diabetes, male C57BL/6 mice were given daily intra-peritoneal injections of STZ diluted in citrate buffer, (40 mg/kg, Sigma-Aldrich, St. Louis, MO) for five consecutive days as described before [32]. Serum glucose concentration in blood obtained from a tail vein, was measured using Prestige LX Smart System Test-strips (Home Diagnostic, Inc., Fort Lauderdale, FL) once a week, beginning 7 days after the last STZ dose. Blood glucose levels of 200 mg/dl (12 mmol/l) or above on two consecutive weeks was considered confirmation of diabetes development. The incidence of diabetes was expressed as the percentage of animals that presented diabetes.

### Cytokine production

Splenocytes were harvested, cultured in RPMI 1640 medium (5 × 10^6^/ml; Life Technologies, Grand Island, NY) and stimulated *in vitro *with 20 μg/ml of recombinant Hsp65 protein or concanavalin A. Cytokine levels in culture supernatants were evaluated 48 hours later by ELISA according to manufacturer instructions. Monoclonal antibodies derived from the clones G281-2626 and MP6-XT3 were used as capture and biotinylated antibodies, respectively, for TNF-α quantification, whereas mAb clones JES5-2A5 and SXC-1 were used to detect IL-10. The detection limit was 15 pg/ml. Antibodies and recombinant cytokines were purchased from Pharmingen (San Diego, CA).

### Analysis of splenic cell populations by flow cytometry

T-cell subsets were determined with mAbs specific for cell surface markers. Phycoerythrin (PE)- labeled mAbs included anti-CD8 mAbs (clone 53-6.7, rat IgG2a) and anti-CD25 mAbs (PC61, rat IgG1). Fluorescein isothiocyanate (FITC)-labeled mAbs were also used: anti-CD4 (clone H129.19); anti-CD103 (M290, rat IgG2a); and anti-CTLA-4 (clone 1B8, hamster IgG). In addition, a Percp-labeled, anti-CD4 mAb (clone RM4-5) was used. Hamster IgG and rat IgG2a, together with rat IgG2b labeled with PE, FITC or Percp, were used as isotype controls. All mAbs were purchased from PharMingen and were used according to manufacturer instructions.

Spleen cells (1 × 10^7^/ml) were initially incubated for 30 min at 4°C with Fc Block (1 μg/10^6 ^cells; PharMingen). The cells were then incubated with the proper mAb (0.75 μg/10^6 ^cells) for 30 minutes at 4°C in total darkness. Lymphocytes were analyzed through flow cytometry using the CellQuest computer program (FACSort; Becton Dickinson, San Jose, CA). Ten thousand events per sample were collected, and double-color fluorescence-activated cell sorter analysis was performed as described elsewhere [33].

### Histology and immunohistochemistry

Five weeks after the final STZ injection, the pancreas were removed and sections (4-5 μm) were stained with hematoxylin-eosin (Merck, Whitehouse Station, NJ). The degree of insulitis was scored using the following scale: 0 = intact islet; 1 = peri-insulitis; 2 = moderate insulitis (< 50% of the islet infiltrated); 3 = severe insulitis (≥ 50% of the islet infiltrated); and 4 = destructive insulitis. At least 20 islets per pancreas were analyzed by two independent examiners. Staining for CD4, CD8, IL-10 and TNF-α was performed on cryostat acetone-fixed pancreatic sections by incubation for 1 hour with either rat anti-mouse primary mAb (Pharmingen) diluted 1:200 in 3% bovine serum albumin (BSA)-PBS, or with rat anti-mouse CD25 biotinylated mAb (Pharmingen) diluted 1:200 in 3% BSA-PBS. This was followed by 1 hour of incubation with biotin-conjugated rabbit anti-rat antibody (Pharmingen). The color was revealed using the 3,3'-diaminobenzidine revelation system (Vector kit; Vector Laboratory, Burlingame, CA). For the control of nonspecific staining, pancreas sections were treated without anti-mouse primary mAb. In addition, the pancreas immunohistochemistry was further quantified by using the image processing program (ImageJ, U.S. National Institutes of Health, Bethesda, Maryland, USA) [34].

### Statistical Analysis

The results are presented as mean ± SD. Statistical significance of diabetes incidence and degree of insulitis was determined by using Fisher's exact test. For ELISA and fluorescence-activated cell sorter results, the statistical significance was determined by One-Way analysis of variance followed by Tukey's test. Values of *P *< 0.05 were considered significant.

## Results

### DNA-HSP65 decreases pancreatic insulitis in STZ induced diabetes

**Figure 1:**
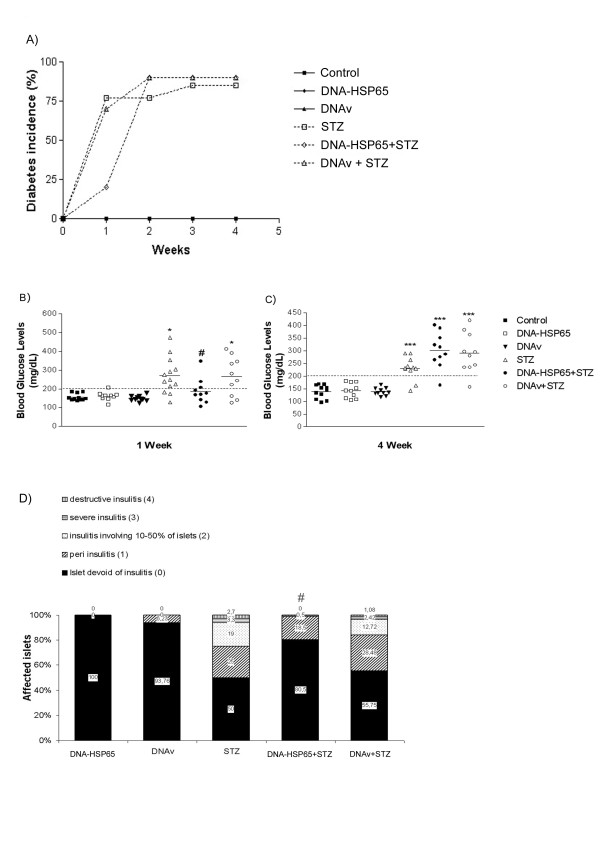
**Effect of DNA-HSP65 on diabetes incidence and insulitis severity**. (**A**) Diabetes incidence of male mice (6-8 weeks old) that were i.m. injected with three 100-μg doses of DNA-HSP65 or DNAv, given at 15-day intervals, after which they were i.p. injected or not with STZ. Control group mice received i.m. injections of PBS. (**B **and **C**) Serum glucose levels were assayed weekly after 5 doses of STZ administration. Additional control groups received only DNA-HSP65 or DNAv. (**D**) Pancreata of C57BL/6 mice were removed for histological analysis. Each group consisted of 10 mice. To quantify islet infiltration, at least 20 islets from three sections per pancreas were examined in a blinded fashion. Statistical significance was determined by ANOVA followed by Tukey's test. *p < 0.05 and ***p < 0.001 versus control group; ^#^p < 0.05 versus STZ groups.

Glucose concentrations were analyzed weekly after the final STZ injection. As shown in Fig. 1A-C, administration of DNA-HSP65 or DNAv did not induce diabetes or significantly altered diabetes incidence induced by STZ in mice. Notably, administration of DNA-HSP65 clearly delayed diabetes development (DNA-HSP65+STZ group) during the first week (Fig. 1A and 1B). Diabetes incidence at this period was 25% in DNA-HSP65+STZ group, compared with 75% in the STZ group (Fig. 1A and 1B). However, four weeks later, diabetes incidence was similar in the three groups that received STZ (Fig. 1A and 1C). The histological pancreas analysis documented in Fig. 1D, clearly demonstrates that previous DNA-HSP65 vaccination determined a protective effect. As expected, mice from the STZ group presented a clear pattern of insulitis, characterized by peri-insulitis (in 25% of the islets), moderate insulitis (in 19%), invasive insulitis (in 3.3%) and destructive insulitis (in 2.7%). A very similar islet damage profile was observed in the DNAv+STZ group. However, DNA-HSP65+STZ group presented considerably less islet injury, characterized by a significantly higher percentage of intact islets, i.e. 81% in comparison to 50% in the STZ group and 56% in DNAv+STZ group. Also, no destructive insulitis was observed in this previously vaccinated group.

### IL-10 but not TNF-α is modulated by DNA inoculation

**Figure 2:**
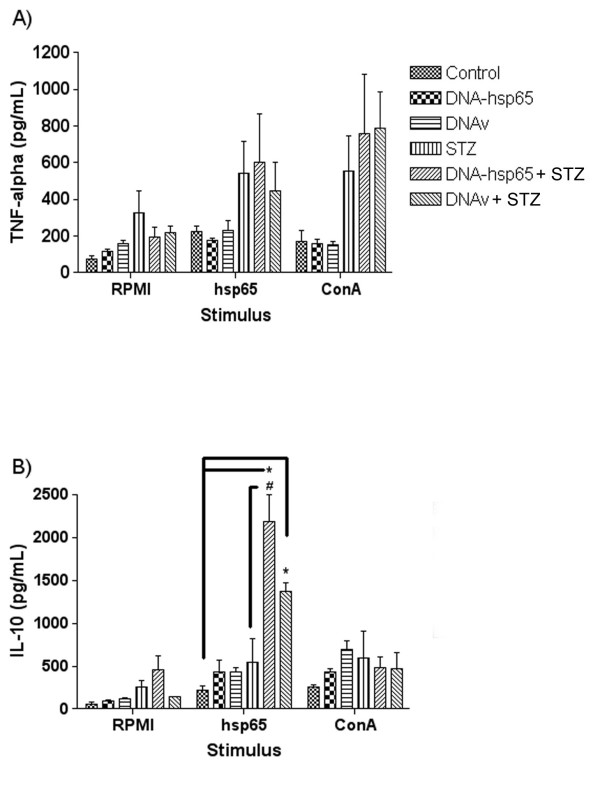
**Cytokine production in STZ injected mice previously vaccinated with DNA-HSP65**. TNF-α (A) and IL-10 (B) were assessed by ELISA in spleen cell cultures stimulated with recombinant Hsp65 and concanavalin A. Statistical significance was determined by ANOVA followed by Tukey's test. *p < 0.05 versus control group and ^#^p < 0.05 versus STZ group.

To understand the prophylactic effect of DNA-HSP65 in the STZ model, we initially analyzed the production of TNF-α and IL-10 in spleen cell cultures. TNF-α levels were higher in STZ, DNAv+STZ and DNA-HSP65+STZ derived splenic cultures stimulated with recombinant Hsp65 or concanavalin A than in non-stimulated cultures, however, there was no significant difference among these three groups (Fig. 2A). Nevertheless, in the cultures from mice injected with DNA (vector and vaccine) before STZ diabetes triggering, the stimulation with rhsp65 determined a very significant increase in IL-10 production. Interestingly, production of IL-10 was significantly higher in vaccinated mice in comparison to the control vector injected group (Fig. 2B).

### DNA-HSP65 activates spleen cells with a regulatory phenotype

**Figure 3:**
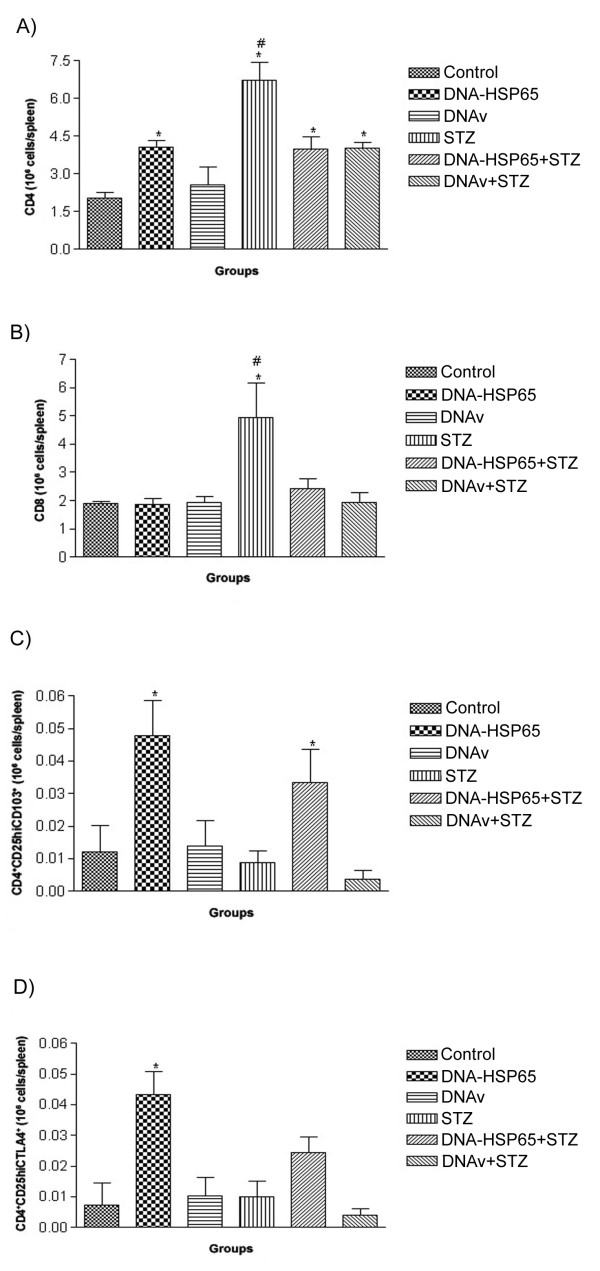
**Assessment of spleen cell phenotype in STZ injected mice after administration of DNA-HSP65**. Five weeks after the STZ injections, 5 × 10^6 ^spleen cells were labeled with anti-CD4, anti-CD8, anti-CD25, anti-CD103 and anti-CTLA-4 mAbs for fluorescence-activated cell sorter analysis. The results were expressed in total number of cells per spleen for CD4^+ ^(A), CD8^+ ^(B) T cells and CD4^+^CD25^+^CD103^+ ^(C) and CD4^+^CD25^+^CTLA-4^+ ^(D) T regulatory cells. Statistical significance was determined by ANOVA followed by Tukey's test. *p < 0.05 versus control group; ^#^p < 0.05 versus STZ groups.

The higher IL-10 production in mice that received DNA before STZ suggested the possible contribution of regulatory T cells in this protection against insulitis. In this context, we initially characterized the populations and phenotypes of T cells in the spleen. Clear differences were observed in the amounts of CD4^+ ^and CD8^+ ^T cells (Fig. 3). Animals that were immunized with DNA-HSP65 presented higher numbers of CD4^+ ^cells but not of CD8^+ ^cells. This alteration was not seen in animals injected with the empty vector. Injection of STZ alone clearly increased the number of both cell types and interestingly prior administration of DNA-HSP65 or DNAv was associated with significantly lower numbers of these cells (Figs. 3A and 3B). The analysis of phenotypic markers indicative of regulatory ability, including CD4^+^CD25^high^CD103^+ ^and CD4^+^CD25^high^CTLA-4^+^, clearly showed that these cells were induced or activated in mice injected with the DNA-HSP65 but not with the empty vector (Figs. 3C and 3D). Moreover, DNA-HSP65 injection followed by STZ clearly increased the number of cells showing the regulatory phenotypic markers in comparison to STZ group. No such up-regulation was observed in the group of mice that received the DNAv before induction of diabetes with STZ. Interestingly, in the spleens of STZ-injected mice, high numbers of CD4^+^CD25^high ^cells were observed (data not shown), although the numbers of CD4^+^CD25^high^CD103^+ ^and CD4^+^CD25^high^CTLA-4^+ ^cells remained at similar levels as the control group (Figs. 3C and 3D).

### Pancreatic islets show decreased CD8^+ ^cell infiltration in DNA-HSP65-vaccinated mice

**Figure 4:**
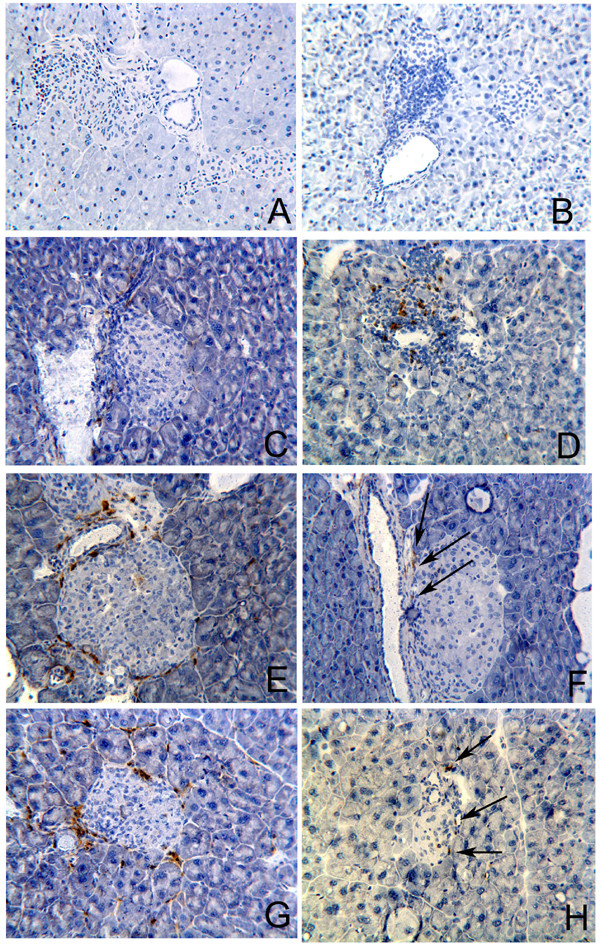
**Influx of CD4^+ ^and CD8^+ ^cells into the pancreatic islets of STZ-injected mice previously immunized with DNA-HSP65**. After three 100-μg doses of DNA-HSP65 or DNAv, given at 15-day intervals, mice were injected with STZ. Control group mice received STZ only (i.p. injections). Pancreatic islets were analyzed 5 weeks after the last STZ injection. Immunostaining for CD4 (A, C, E and G) and CD8 receptors (B, D, F and H). Unspecific staining control (A and B). STZ group mice (C and D), DNA-HSP65+STZ group mice (E and F) and DNAv+STZ group mice (G and H). Quantitative analyses were performed by image processing program (ImageJ). Original magnification, ×20.

We next considered the possibility that regulatory cells were migrating to the pancreas to block insulitis. In the STZ group there was pronounced pancreatic infiltration by CD8^+ ^cells (Fig. 4D) but not by CD4^+ ^cells (Fig. 4C). By evaluating the stained area for CD8^+ ^and CD4^+ ^cells we observed that CD8^+ ^cell staining was 2 fold higher than CD4^+ ^cell in STZ group (2,45% and 1,20% of stained area for CD8 and CD4, respectively). In DNA-HSP65+STZ and DNAv+STZ groups of mice, this profile was dramatically changed showing a marked infiltration by CD4^+ ^cells (Figs. 4E and 4G, respectively) associated with a quite discrete infiltration by CD8^+ ^cells (Figs. 4F and 4H, respectively). These groups presented CD4^+ ^cell staining three fold higher than CD8^+ ^cell staining (2,40% and 0,79% for CD4 and CD8, respectively, in DNA-HSP65+STZ group and, 2,21% and 0,71% for CD4 and CD8, respectively, in DNAv+STZ group).

### Pancreatic islets fromDNA-HSP65 injected mice show increased IL-10 production

**Figure 5:**
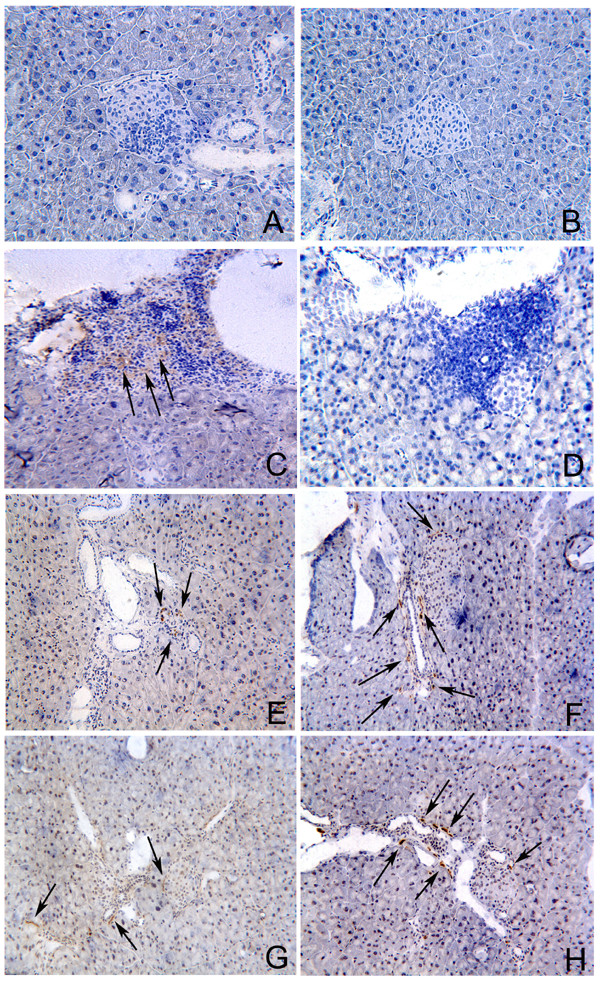
**TNF-α and IL-10 cytokine production in the pancreatic islets of DNA-HSP65+STZ and DNAv+STZ group mice**. After three 100-μg doses of DNA-HSP65 or DNAv, given at 15-day intervals, they were inoculated with STZ. Control group mice received STZ only (i.p. injections). Pancreatic islets were analyzed from 5 weeks after the last STZ injection. Unspecific staining control (A and B). Levels of TNF-α (A, C, E and G) and IL-10 (B, D, F and H) were evaluated in STZ group mice (C and D), DNA-HSP65+STZ group mice (E and F) and DNAv+STZ group mice (G and H). Quantitative analyses were performed by image processing program (ImageJ). Original magnification, ×20.

Since a higher number of CD4^+ ^cells and a lower number of CD8^+ ^cells were present in the islets of DNA-HSP65-injected and DNAv-injected mice in relation to STZ group, we supposed that CD4^+ ^cells with a regulatory phenotype could be involved in down modulation of diabetes development. To confirm the hypothesis that an anti-inflammatory and regulatory cytokine was involved in protection against insulitis in this model, we investigated the local production of IL-10 and TNF-α. In the pancreatic islets of STZ-injected mice there was abundant local TNF-α production and no IL-10 production (Figs. 5C and 5D, respectively). Analysis of stained area showed that 1,25% of total area was staining for TNF-α. The DNA-HSP65+STZ and DNAv+STZ groups also showed local TNF-α production. Labeled areas were 0,31% and 0,56% for vaccinated and vector injected groups, respectively (figs. 5E and 5G). However, DNA-HSP65+STZ and DNAv+STZ groups of mice presented a marked IL-10 production in the islets. The IL-10 stained area in DNA-HSP65+STZ and DNAv+STZ was 1,52% and 1,36%, respectively. This represented an increase of 5 and 2,5-folds, respectively, when compared with TNF-α labeled area (Figs. 5F and 5H).

## Discussion

The present study clearly demonstrated that previous vaccination with DNA-HSP65 did not aggravate or accelerate diabetes development induced by STZ. In addition, this genetic construction triggered a beneficial effect characterized by a delay in diabetes incidence, a higher percentage of intact islets and also absence of destructive insulitis. The last two effects were also determined by the empty vector, even though they were much less accentuated.

This protective effect was clearly associated with an immunomodulatory activity over cytokines and T cell subsets in both, spleen and islets of Langerhans. TNF-α levels induced in vitro by rhsp65 or ConA were similarly high in STZ, DNA-HSP65+STZ and DNAv+STZ groups. However, the local production of TNF-α in the islets was clearly down modulated by both, DNAv and DNA-HSP65. Mice groups injected with DNAv and DNA-HSP65 before STZ also produced significantly higher IL-10 levels in comparison to STZ group. It was also striking the finding that IL-10 levels were significantly more elevated in the DNA-HSP65 group than in the corresponding DNAv+STZ control. This IL-10 profile in the periphery (spleen) was coincident with a corresponding pattern at the pancreas, i.e., IL-10 absence in STZ group and clear IL-10 production in DNA injected mice. These findings are consistent with the widely accepted role of IL-10 as a suppressive cytokine involved in autoimmunity control. For example, Zhang et al., showed that systemic administration of IL-10 plasmid DNA alleviated insulitis and reduced disease incidence in experimental autoimmune diabetes [35]. Moreover, oral or nasal mucosal immunization with *Mycobacterium *Hsp65 that resulted in protection against atherosclerosis was also related to IL-10 production [36]. Although IL-10 production was stimulated by both, DNA-HSP65 and DNAv, it is important to emphasize that only animals immunized with DNA-HSP65 presented an increased number of CD4^+^CD25^high^CD103^+ ^cells. Zanin-Zhorov et al., reported that the human 60-kDa heat shock protein (Hsp60) acts as a co-stimulator of human regulatory T cells (Treg), both CD4+CD25^intermediate ^and CD4+CD25^high^, via innate TLR2 signaling. They observed that the treatment of Treg with Hsp60 or its peptide p277 before anti-CD3 activation significantly enhanced the ability of relatively low concentrations of Treg cells to down-regulate CD4+CD25- or CD8+ target T cells, detected as inhibition of target T cell proliferation and IFN-γ and TNF-α secretion. Interestingly these Hsp60-treated Treg cells suppressed target T lymphocytes by both, cell-to-cell contact and secretion of TGF-β and IL-10 [37]. It is possible therefore that DNA-HSP65 immunization is also priming Hsp65 antigen-specific B and/or T lymphocytes, including Treg cells. These data reinforce our finding that a specific immune response also played a significant role in the protection against diabetes since administration of DNA-HSP65 was associated with higher percentage of intact islets than DNAv, despite IL-10 elicitation by both DNA preparations.

The relevance of the regulatory role of CD4^+^CD25^+ ^T cells has been described in different kinds of experimental diabetes models. In a CD8^+ ^T cell-mediated model of type 1 diabetes, Green et al., showed that CD4^+^CD25^+ ^T cells avoided pancreatic β cell destruction [38] by preventing intra-islet differentiation of CD8^+ ^T cells into cytotoxic T lymphocyte effectors [38]. Intranasal administration of islet auto-antigens in mice also elicited antigen-specific CD4^+^CD25^+ ^Treg cells able to prevent development of type 1 diabetes [39]. In agreement with these findings, our results show that a higher number of spleen cells with this regulatory phenotype was present in DNA-HSP65-injected mice. Interestingly, the number of CD4^+^CD25^high^CD103^+ ^and CD4^+^CD25^high^CTLA-4^+ ^cells in the spleen of the DNA-HSP65-injected mice was significantly higher than in those injected only with STZ. In addition, CD8^+ ^cell infiltration was reduced and levels of IL-10 were higher in the pancreatic islets of DNAv-injected mice, suggesting that DNA-HSP65 injection activated a regulatory mechanism, characterized by IL-10 production that inhibited the infiltration of inflammatory cells into the pancreas. This regulatory ability of DNA-HSP65 and its association with higher IL-10 production was already described by our group in experimental arthritis and also in NOD mice [29,30,40]. Together these findings are highly supported by the well accepted immunoregulatory ability of microbial hsps [41]. In this context it is tempting to suggest that HSP65 is, at least partially, responsible for the islet recovery and reversal of murine type 1 diabetes observed in NOD mice after Complete Freund's Adjuvant administration [42,43].

An interesting and useful challenge for the future is to unravel the exact immunological scenario that will favor maximal protection against tuberculosis or against autoimmunity.

## Conclusion

In conclusion, the data presented in this study encourage us to invest in the regulatory potential of the DNA-HSP65 construct. Our findings have important implications for the development of new immune therapy strategies to combat autoimmune diseases.

## Competing interests

The authors declare that they have no competing interests.

## Authors' contributions

All authors have read and approved the final version of this manuscript. RSJ, AS, DL and PS carried out laboratory work, collected and analysed the data and conducted the transfer and interpretation of the data for final preparation of the manuscript. Statistical analysis was carried out by RSJ. AS, VB, ACC and CS participated in writing the manuscript.
